# Analysis of Global and Local DNA Methylation Patterns in Blood Samples of Patients With Autism Spectrum Disorder

**DOI:** 10.3389/fped.2021.685310

**Published:** 2021-10-05

**Authors:** María Victoria García-Ortiz, María José de la Torre-Aguilar, Teresa Morales-Ruiz, Antonio Gómez-Fernández, Katherine Flores-Rojas, Mercedes Gil-Campos, Pilar Martin-Borreguero, Rafael R. Ariza, Teresa Roldán-Arjona, Juan Luis Perez-Navero

**Affiliations:** ^1^Maimónides Biomedical Research Institute of Córdoba (IMIBIC), Córdoba, Spain; ^2^Department of Genetics, University of Córdoba, Córdoba, Spain; ^3^Reina Sofía University Hospital, Córdoba, Spain; ^4^Department of Pediatrics, Reina Sofia University Hospital, University of Córdoba, Córdoba, Spain; ^5^Pediatric Metabolism Unit, Reina Sofia University Hospital, University of Córdoba, Córdoba, Spain; ^6^Physiopathology of Obesity and Nutrition Networking Biomedical Research Center (CIBEROBN), Córdoba, Spain; ^7^Department of Child and Adolescent Clinical Psychiatry and Psychology, Reina Sofia University Hospital, Córdoba, Spain; ^8^Biomedical Research Center–Rare Diseases (CIBERER), Carlos III Health Institute, Madrid, Spain

**Keywords:** autism spectrum disorder, neurodevelopmental regression, DNA methylation patterns, NCAM, NGF

## Abstract

The goal of this investigation was to determine whether there are alterations in DNA methylation patterns in children with autism spectrum disorder (ASD).

**Material and Methods:** Controlled prospective observational case-control study. Within the ASD group, children were sub-classified based on the presence (AMR subgroup) or absence (ANMR subgroup) of neurodevelopmental regression during the first 2 years of life. We analyzed the global levels of DNA methylation, reflected in *LINE-1*, and the local DNA methylation pattern in two candidate genes, Neural Cell Adhesion Molecule (NCAM1) and Nerve Growth Factor (NGF) that, according to our previous studies, might be associated to an increased risk for ASD. For this purpose, we utilized blood samples from pediatric patients with ASD (*n* = 53) and their corresponding controls (*n* = 45).

**Results:** We observed a slight decrease in methylation levels of *LINE-1* in the ASD group, compared to the control group. One of the CpG in *LINE-1* (GenBank accession no.X58075, nucleotide position 329) was the main responsible for such reduction, highly significant in the ASD subgroup of children with AMR (*p* < 0.05). Furthermore, we detected higher *NCAM1* methylation levels in ASD children, compared to healthy children (*p* < 0.001). The data, moreover, showed higher *NGF* methylation levels in the AMR subgroup, compared to the control group and the ANMR subgroup. These results are consistent with our prior study, in which lower plasma levels of *NCAM1* and higher levels of *NGF* were found in the ANMR subgroup, compared to the subgroup that comprised neurotypically developing children.

**Conclusions:** We have provided new clues about the epigenetic changes that occur in ASD, and suggest two potential epigenetic biomarkers that would facilitate the diagnosis of the disorder. We similarly present with evidence of a clear differentiation in DNA methylation between the ASD subgroups, with or without mental regression.

## Introduction

Autism spectrum disorder (ASD) is a severe neurodevelopmental disorder featuring variable but pronounced deficits in communication and social interaction, and is associated to multiple genetic risk factors (1, 2). Environmental (viral infection, parental age, diet) and epigenetic factors (DNA methylation, histone modification and microRNA) would act on some predisposing genetic factors (3). However, although epigenetic mechanisms, transcriptome profiles, and environmental factors have been suggested to be implicated, no clear pathogenesis mechanisms have been yet identified in ASD (4–12).

One of the best-known epigenetic marks is the 5-methylcytosine (5-mC), generated by the methylation at C5 of cytosine in symmetric CG contexts. A high proportion of regulatory regions, such as promoters and enhancers, contains areas with high density of CpG dinucleotides (called “CpG islands”) that are usually unmethylated, and whose methylation is generally associated to transcriptional silencing (13). Extensive experimental evidence supports that DNA methylation is a crucial step during brain development, and plays a key role in processes including synaptic plasticity, learning, memory, or cognitive decline (14).

Unlike mutations, epigenetic changes are potentially reversible, which has opened new pathways in the study of some diseases. Nowadays, it is accepted that ASD has a strong epigenetic component (14–19). Several genome-wide studies revealed multiple alterations in DNA methylation patterns in the brains of ASD individuals (20, 21). The largest meta-analysis study using peripheral blood samples from about 800 autistic individuals revealed that 55 of examined CpG sites were associated to ASD (22). Nevertheless, the main regions analyzed in these studies are the CpG islands of the protein coding regions, whereas DNA methylation in the genomic non-coding regions, which are often implicated in genetic regulation pathways, has not yet been examined. On the other hand, a significant fraction of the mutations associated to an increased risk of ASD affects transcriptional factors or chromatin-modifying proteins (23). Genetic mutations of the MECP2 gene related to autism spectrum disorders have been recently identified (4). MECP2 is a master epigenetic regulator that exhibits high levels of expression in the brain. However, a reduced expression of MECP2, associated to hypermethylation of its promoter, has been described in the frontal cortex of patients with ASD (8). Understanding the epigenetic changes involved in ASD can also help to identify subgroups of patients, allowing a better stratification for the implementation of pharmacological and/or behavioral therapies.

Long interspersed element-1 (*LINE-1*) is an autonomous transposable element that makes up roughly 17% of the human genome where it remains active (24). It is possible for retrotransposition of *LINE-1* into a new position of the genome to cause duplication, deletion, or insertion at the target site, triggering genomic instability and changes in gene expression (25). Although the process by which *LINE-1* transcription and retrotransposition is regulated remains indeterminate, mounting evidence propose that epigenetic pathways, such as DNA methylation and histone modifications, are implicated in the retrotransposition of *LINE-1* and could have an impact on the target genes expression (26). There have been reports of altered DNA methylation levels and patterns of CpG residues in *LINE-1* promoter regions in many diseases, including ASD (22, 26).

Other authors have posited that both a proinflammatory condition and an alteration in adhesion molecules in the early stages of neurodevelopment may play a role in the pathophysiology of ASD. Our group has recently published a study on the plasma levels of particular adhesion molecules and growth factors in patients with ASD compared to healthy children. When sub-classifying according to the presence or absence of neurodevelopmental regression in children with ASD, lower plasma levels of the Neural Cell Adhesion Molecule (NCAM1) and higher levels of Nerve Growth Factor (NGF) were observed in the subgroup of autistic children without mental regression, compared to the levels obtained in the mental regression subgroup and the control group (27).

Based on evidence of close implication of loci-specific DNA methylation in the pathophysiology of neurological disorders including ASD (20, 21), and previous demonstration of an association between the epigenetic regulation of *LINE-1* and its effects in ASD pathobiology (26), the present study aims to compare global methylation levels in blood samples of pediatric patients with ASD and healthy children, and to examine the DNA methylation status of *NCAM1* and *NGF, two* genes that we have previously shown to be deregulated in autistic spectrum disorder. Here, we report the results of a global and local DNA methylation analysis from a pediatric sample with ASD compared to normally developing children.

## Materials and Methods

### Subjects

This was a controlled prospective observational case-control study. The patients with ASD were recruited from the Department of Child and Adolescent Clinical Psychiatry and Psychology at the Reina Sofia University Hospital. Some of the patients (those aged 2–3) were assimilated into the study at the time of their ASD diagnosis, while the older patients (those aged 3–6) were selected from among children who had already been given diagnoses at the same unit. The diagnosis of ASD was based on the clinical judgment of professionals specializing in identifying the unique developmental profile associated with subjects with ASD. The diagnoses were based on the information obtained from semi-structured clinical development interviews, and psychological and behavioral tests that have been internationally acknowledged to be reliable and valid for this purpose. Two clinical psychologists, a psychiatrist and an occupational therapist with extensive clinical experience and training in diagnostic tests for research in ASD performed the diagnoses, employing the criteria established in DSM-5 (28). In addition, two pediatricians reviewed the subjects' medical histories and conducted an examination of all the children.

Children with ASD presenting with other known neurological, metabolic or genetic diagnoses were excluded from the study, and the same applied to children undergoing medical treatments for autism-related behavioral comorbidities that might interfere with the results, such as sedatives, muscle relaxants or similar. In addition, a control group of normally developing children was recruited, matched to the ASD group in terms of gender and age.

Within the ASD group, children were sub-classified based on the presence or absence of neurodevelopmental regression occurring during the first 2 years of life. This was assessed by means of a five-item questionnaire in accordance with the guide used by the ADI-R clinical interview for the evaluation of this process (29). Children with ASD who obtained a score equal to or greater than three were included in the mental regression subgroup (AMR: Autism Mental Regression), while those with a score inferior to this cut-off value were included in the non-mental regression subgroup (ANMR: Autism Non-Mental Regression) (27).

The study was approved by the Clinical Research and Bioethics Committee at the Reina Sofia University Hospital, conforming to the fundamental principles established in the Declaration of Helsinki (latest version 2013), supplemented by the Declaration of Taipei (2016).

### Standardized Diagnostic Measurements and Assessment of ASD Severity

All the cases of ASD were selected after carrying out the Checklist for Autism in Toddlers (M-CHAT). Subsequently, a comprehensive clinical history of each child was taken. The Autism Diagnostic Observation Schedule, Second Edition (ADOS-2), was used in cases of suspected ASD. Each of the following tests was also administered: the Autism Diagnostic Interview-revised (ADI-R), the Pervasive Developmental Disorders Behavior Inventory™ (PDDBI), the Childhood Autism Rating Scale test (CARS), the Battelle Developmental test, and the Strengths and Difficulties Questionnaire (SDQ). These tests enabled the clinical team to diagnose ASD with more certainty.

None of the ASD cases selected for the present study exhibited any other associated pathology (seizures, or other neurological, metabolic or genetic diseases). Each patient was clinically assessed with complementary explorations. The children also underwent a genetic study (karyotype and microarrays) to detect secondary or syndromic ASD. None of the patients required medication to treat behavioral disorders or aggression.

### Sample Collection, DNA Extraction and Bisulfite Treatment of the DNA

After overnight fasting, peripheral whole blood samples were collected from participants *via* antecubital vein into 6-ml blood collection tubes containing EDTA. After centrifugation at 3,500 g for 10 min, plasma was divided into aliquots and processed within 2 h from sampling, and then frozen at −80°C until analysis. Blood count and a general biochemical analysis were performed to confirm the absence of other diseases.

Genomic DNA was extracted from 2 mL of plasma with the QIAamp DNA Blood kit (QIAGEN), following the manufacture's protocol, and quantified in a NanoDrop ND-1,000 spectrophotometer (Nano-Drop Technologies). Bisulfite conversion of isolated DNA (500 ng) was performed with the EZ DNA Methylation Gold Kit (Zymo Research), according to the manufacturer's instructions.

### PCR and Pyrosequencing

Utilizing the Immolase DNA Polymerase (Bioline) 50 ng of bisulfite, converted DNA were added to the PCR reaction, following the manufacturer's recommendations. Table 1 shows the forward and reverse primer sequences for each gene. The PCR product was subjected to agarose gel electrophoresis to test for the presence of a single PCR product. 15 μL of the verified biotinylated PCR product was used for each sequencing assay. DNA pyrosequencing was performed in a PyroMark Q24 instrument (Qiagen), following the manufacturer's guidelines, and subsequent methylation analysis was determined with the PyroMark Q24 Software (Qiagen). Sequencing primers for each gene are listed in Table 1. In the light of the literature (30–33) and recent results from this group of children (27), two loci previously linked to ASD (*NCAM1* and *NGF*) were selected for methylation analysis. Primers for *NCAM1* were designed to amplify a 3′UTR region (UCSC region chr11:112,965,249–112,965,403) that included CpG positions for which changes in methylation levels have been previously described (34). Primers for *NGF* were obtained from Qiagen to amplify a region at the 5′UTR, near the non-transcribed first exon of the gene (UCSC region chr1:115,880,563–115,880,774) (Table 1).

**Table 1:** List of primers used for pyrosequencing.

**Gene**	**Primer**	**Sequence (5^**′**^-3^**′**^)**	**Length (bp)**	**Accession no. (Gene ID)**	**Annealing *T*_**m**_ (^**°**^C)**
*LINE-1*	Forward	TTTTGAGTTAGGTGTGGGATATA	146	NM_019079 (54596)	50
	Reverse	[BTN] AAAATCAAAAAATTCCCTTTC			
	Sequencing	AGTTAGGTGTGGGATATAG			
*NCAM1*	Forward	TATTTTTGTGTTTTTTTGGGGGTTAGATTA	154	NM_181351 (4684)	55
	Reverse	[BTN] CCCAACTATACAATCTTCTCTACTTCAT			
	Sequencing	GGGGTTAGATTATTTTTTGAT			
*NGF*	Forward	Hs_NGF_01_PM PyroMark CpG assay (PM00002618)	211	NM_002506 (4803)	58
	Reverse				
	Sequencing				

*LINE-1, Long interspersed element-1; NCAM1, Neural Cell Adhesion Molecule; NGF, Nerve Growth Factor*.

### Statistical Analysis

The sample size for this study was determined based on the most relevant genome-wide and local DNA methylation studies on ASD using published data results (18, 24, 26). GraphPad Prism 6 Software was used to perform the statistical analyses. The data are expressed as mean ± SD (95% confidence intervals), median (IQR) or absolute (relative frequencies). For data that fit a normal distribution (Shapiro-Wilk normality test) *t*-tests were used. For data not normally distributed, the non-parametric Mann-Whitney test were applied. Categorical variables were evaluated using the χ^2^-test or the Fisher exact test. To assess the methylation differences between healthy control group and ASD subgroups, in CpG positions together or individually, unpaired *t*-test and two-way ANOVA model with Tukey's HSD *post-hoc* tests were used Correlations between methylation levels and the scores obtained from the various tests performed were carried out using the Spearman's p (rho). Receiver operating characteristics (ROC) analysis was conducted to calculate the area under the curve (AUC). All the tests were two-tailed, and a *p* < 0.05 was considered statistically significant.

## Results

### Global Methylation Analysis in Autism Spectrum Disorder

Fifty-four children with ASD and 45 healthy children were included in the study. The principal demographic and test results for the diagnosis are shown in Table 2. Within the ASD group, there were 20 children with AMR and 33 with ANMR; one child could not be classified in these subgroups because he was adopted and there was no previous clinical information.

**Table 2:** Demographic and anthropometric data in children with autism spectrum disorders compared to controls.

		**ASD**	**Control**	***p***
		**(n:50)**	**(n:45)**	
Age (months)		43.76 ± 11,2	48.81 ± 18.33	0.117
Gender (male)		41 (82%)	38 (84%)	0.472
Weight (kg)		16.97 ± 3.51	17.06 ± 4.5	0.951
Battelle test	AMR	47.05 ± 10.33		0.002
	ANMR	60.96 ± 13.29		
CARS test	AMR	35.9 ± 8.12		0.009
	ANMR	30.6 ± 6.11		
PDBBI test	AMR	53.93 ± 10.39		0.023
	ANMR	46.13 ± 10.47		

*ASD, autism spectrum disorders; AMR, autism mental regression; ANMR, autism non-mental regression; PDDBI, Pervasive Developmental Disorders Behavior Inventory; CARS, Childhood Autism Rating Scale*.

**Figure 1:**
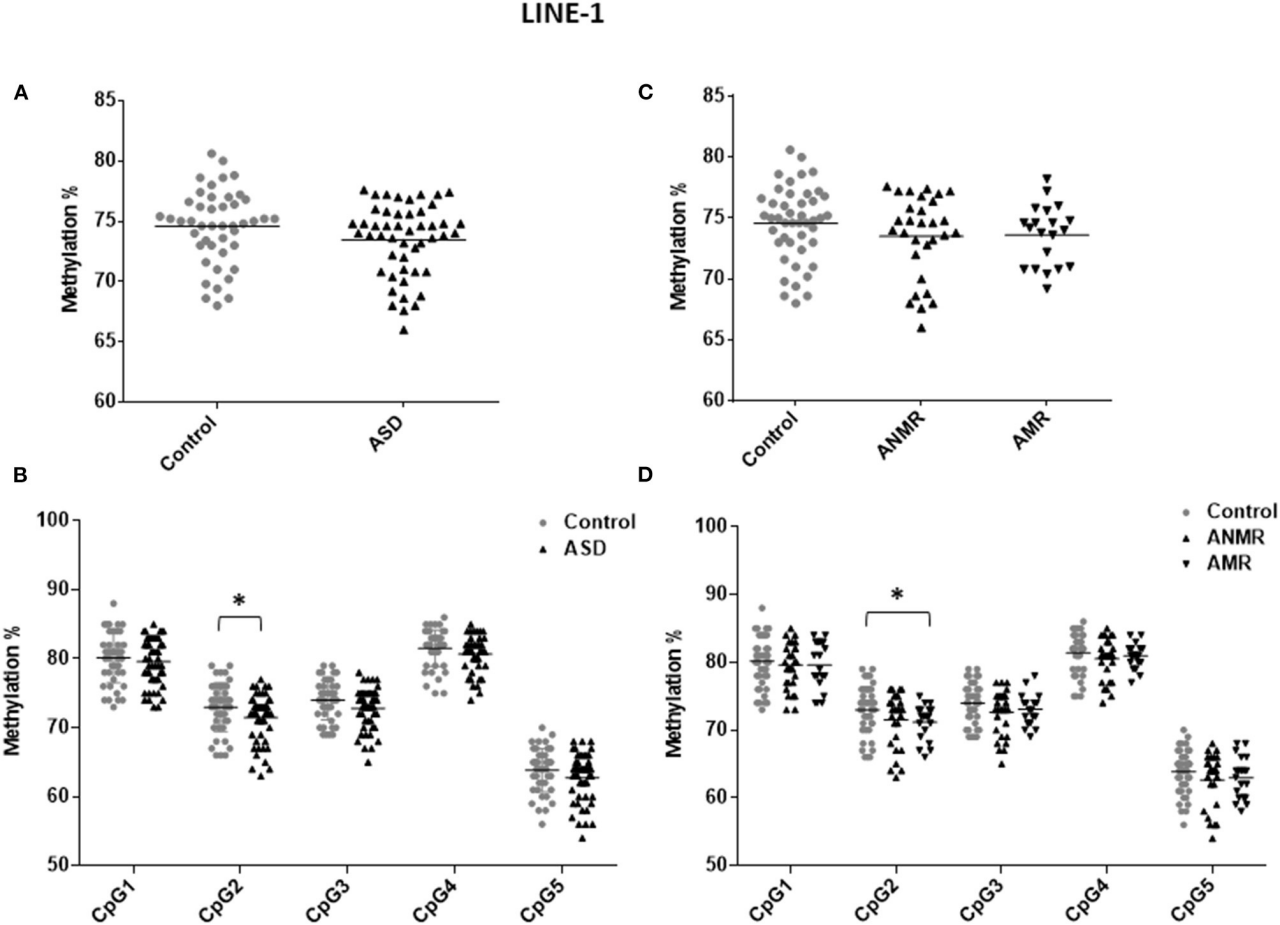
Scatter plot showing comparisons of methylation status of LINE-1 in children with autism spectrum disorders (ASD) compared to controls. **(A,B)** Methylation levels and differentially methylated sites (CpG1–CpG5) in the LINE-1 sequence in ASD group and healthy control group. **(C,D)** Methylation levels and differentially methylated sites in the LINE-1 sequence in ASD group subdivided as follows: ANMR (non-mental regression group), AMR (mental regression group), and a healthy control group. The dark bars represent the mean values of each group. Asterisks indicate statistically significant differences (*P ≤ 0.05) compared to control group.

Because *LINE-1* retrotransposon represents a considerable portion of the human genome, the methylation level of *LINE-1* reflects the global DNA methylation status (24). To determine whether global methylation levels were altered in ASD, 53 blood samples from children with ASD and 45 from the control group were drawn to isolate to gDNA. Bisulfite pyrosequencing was performed analyzing 5 CpG positions of the repetitive element *LINE-1*. Three samples from ASD patients gave inconclusive results, so they were removed from the *LINE-1* methylation analysis. Methylation of *LINE-1* was slightly reduced in the group of children with ASD compared to the control group (73.45 ± 0.43 vs. 74.56 ± 0.45) (Figures 1A,C). When each studied CpG of the *LINE-1* fragment was analyzed separately, position 2 (GenBank accession no.X58075, nucleotide position 329, complementary strand) was the main responsible for the observed decrease in *LINE-1* methylation levels in the ASD group (Figure 1B and Table 3). Moreover, when analysis was performed with patients with AMR and AMNR, methylation levels of CpG 2 from *LINE-1* in the AMR subgroup was significantly lower (71.16 ± 0.60), compared to the healthy control group (72.98 ± 0.52) (Figure 1D and Table 3). Although methylation levels at position 2 of *LINE*-*1* in the ANMR subgroup were also lower than in the control group, this difference was not statistically significant.

**Table 3:** Pyrosequencing results of LINE-1 showing percent change in methylation in ASD group, ANMR and AMR subgroups compared to healthy control group.

	**LINE-1**					
	**Control vs. ASD**		**Control vs. ANMR**		**Control vs. AMR**	
	**Percent change**	***p*-value**	**Percent change**	***p*-value**	**Percent change**	***p*-value**
	**in methylation**		**in methylation**		**in methylation**
All CpG	1.11	0.078	1.06	0.16	0.95	0.15
CpG site 1	0.56	0.49	0.55	0.51	0.56	0.62
CpG site 2	1.49^*^	0.05	1.49	0.15	1.82^*^	0.03
CpG site 3	1.15	0.08	1.34	0.13	0.88	0.23
CpG site 4	0.81	0.06	0.84	0.10	0.42	0.15
CpG site 5	1.07	0.17	1.19	0.28	0.89	0.24

*ASD, autism spectrum disorders; AMR, autism mental regression; ANMR, autism non-mental regression*.

* *P ≤ 0.05 with statistically significant changes highlighted in bold*.

As expected from slight changes in *LINE-1* methylation levels, no clinical correlations were observed between the methylation status of *LINE-1* and any of the tests performed to evaluate the diagnosis, severity, and the regressive aspects of the disorder (Supplementary Table 4).

### Methylation Status Analysis of *NCAM1* and *NGF* in ASD

**Figure 2:**
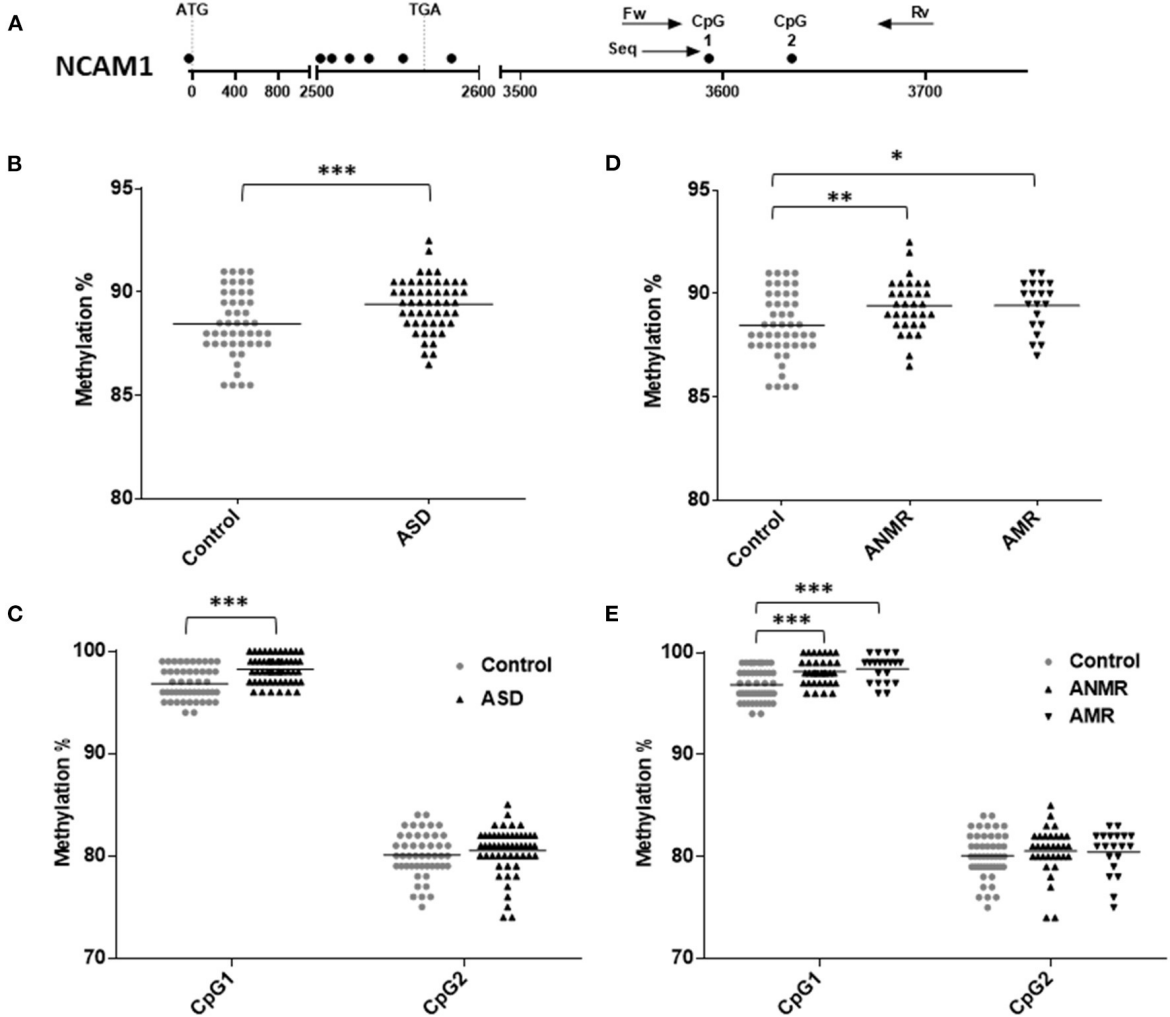
Scatter plot showing comparisons of methylation status of *NCAM1* in children with autism spectrum disorders (ASD) compared to controls. **(A)** Schematic diagram of analyzed gene. Each circle represents a CpG dinucleotide. Position of ATG and TGA codon are indicated. Arrows show the location of pyrosequencing primers. **(B,C)** Methylation levels and differentially methylated sites (CpG1 and CpG2) in the *NCAM1* gene in ASD group and healthy control group. **(D,E)** Methylation levels and differentially methylated sites in the *NCAM1* gene in ASD group subdivided as follows: ANMR (non-mental regression group), AMR (mental regression group), and a healthy control group. The dark bars represent the mean values of each group. Asterisks indicate statistically significant differences (*P < 0.05; **P < 0.01; ***P < 0.001) compared to control group.

**Figure 3:**
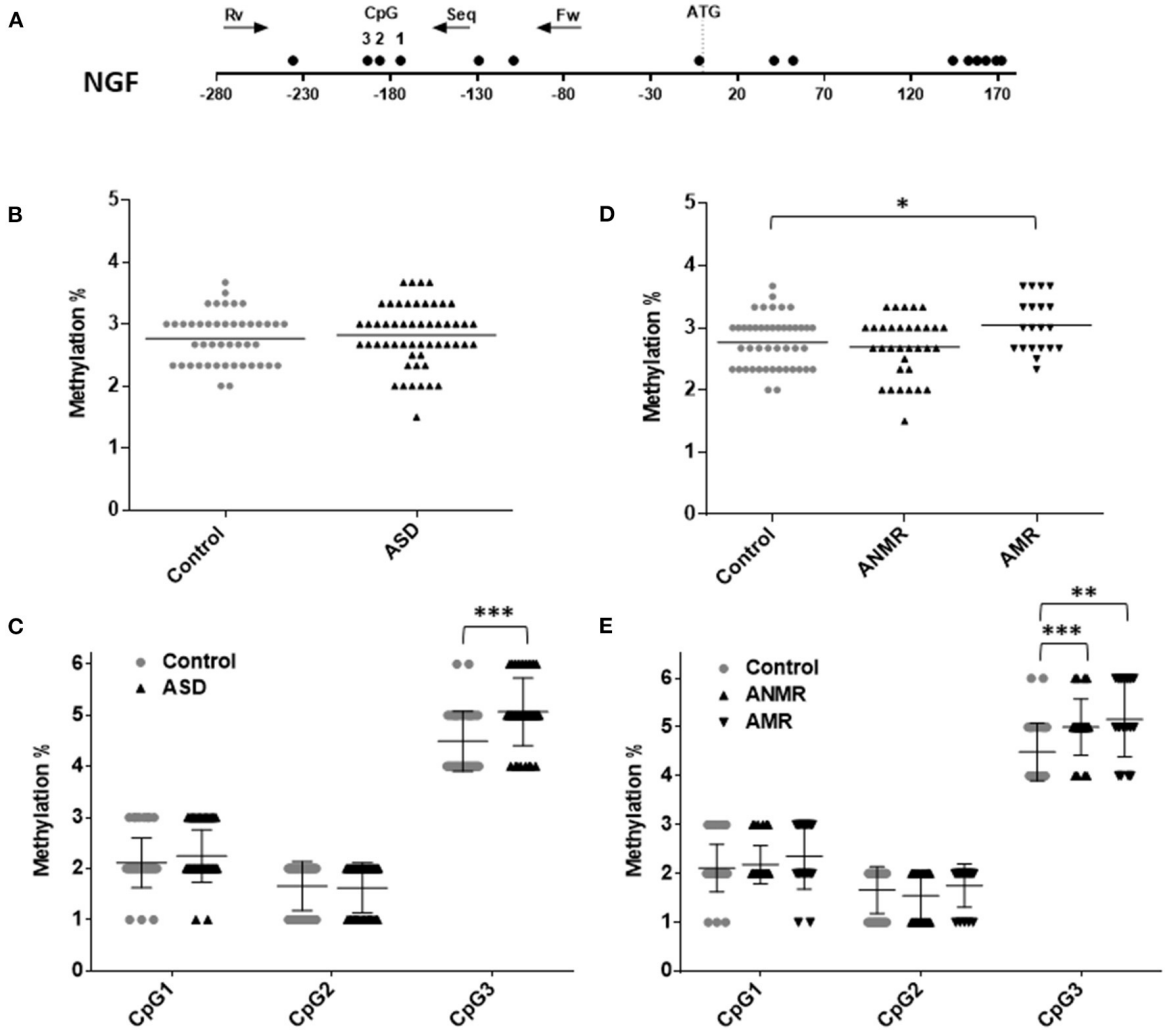
Scatter plot showing comparisons of methylation status of *NGF* in children with autism spectrum disorders (ASD) compared to controls. **(A)** Schematic diagram of analyzed gene. Each circle represents a CpG dinucleotide. Position of ATG codon is indicated. Arrows show the location of pyrosequencing primers. **(B,C)** Methylation levels and differentially methylated sites (CpG 1, 2, and 3) in the *NGF* gene in ASD group and healthy control group. **(D,E)** Methylation levels and differentially methylated sites in the *NGF* gene in ASD group subdivided as follows: ANMR (non-mental regression group), AMR (mental regression group), and a healthy control group. The dark bars represent the mean values of each group. Asterisks indicate statistically significant differences (^*^P < 0.05; ^**^P < 0.01; ^***^P < 0.001) compared to control group.

DNA methylation status was analyzed for each gene (Figures 2A, 3A), both in the ASD group and in the subgroups ANMR and AMR separately. Pyrosequencing results showed a significant increase in *NCAM1* methylation levels in the ASD children and in each subgroup, ANMR and AMR, compared to healthy children (Figures 2B,D and Table 4). Specifically, 70% of ASD individuals presented *NCAM1* methylation levels above the mean, with no distinction between AMR and ANMR. In the control group of healthy individuals, only 42% had higher-than-mean *NCAM1* methylation levels. In addition, CpG 1 in *NCAM1* (UCSC location chr11:112,965,292) was the position that suffered this notable increase in methylation, especially in the AMR subgroup (Figures 2C,E).

**Table 4:** Pyrosequencing results of NCAM1 showing percent change in methylation in ASD group, ANMR and AMR subgroups compared to healthy control group.

	**NCAM1**					
	**Control vs. ASD**		**Control vs. ANMR**		**Control vs. AMR**	
	**Percent change**	***p*-value**	**Percent change**	***p*-value**	**Percent change**	***p*-value**
	**in methylation**		**in methylation**		**in methylation**
All CpG	−0.96^***^	0.0009	−0.96^**^	0.005	−0.97^*^	0.016
CpG site 1	−1.41^***^	<0.0001	−1.31^***^	0.0003	−1.57^***^	0.0002
CpG site 2	−0.43	0.16	−0.46	0.19	−0.37	0.34

*ASD, autism spectrum disorders; AMR, autism mental regression; ANMR, autism non-mental regression; NCAM1, Neural Cell Adhesion Molecule*.

* *P ≤ 0.05*;

** *P < 0.01*;

*** *P < 0.001 with statistically significant changes highlighted in bold*.

On the other hand, no differences were observed in the levels of *NGF* methylation between the ASD group and the healthy group (Figure 3B). Concretely, lower-than-mean levels of *NGF* methylation were detected in 47% of children diagnosed with ASD and in 53% of neurotypically developing children. Within the ASD group, the AMR subgroup had higher-than-mean *NGF* methylation levels than both the ANMR subgroup and the normally developing children (Figure 3D). Methylation analysis of each CpG revealed that the CpG at position 3 of the analyzed region (UCSC location chr1: 115,880,705) showed a higher level of methylation in children diagnosed with ASD, with or without mental regression (Figures 3C,E and Table 5).

**Table 5:** Pyrosequencing results of NGF showing percent change in methylation in ASD group, ANMR and AMR subgroups compared to healthy control group.

	**NGF**					
	**Control vs. ASD**		**Control vs. ANMR**		**Control vs. AMR**	
	**Percent change**	***p*-value**	**Percent change**	***p*-value**	**Percent change**	***p*-value**
	**in methylation**		**in methylation**		**in methylation**
All CpG	−0.05	0.20	0.04	0.81	−0.28^**^	0.029
CpG site 1	−0.13	0.21	−0.07	0.61	−0.24	0.09
CpG site 2	0.04	0.83	0.11	0.35	−0.09	0.57
CpG site 3	−0.49^***^	0.0004	**−0.51**	0.0008^***^	−0.67^**^	0.001

*ASD, autism spectrum disorders; AMR, autism mental regression; ANMR, autism non-mental regression; NGF, Nerve Growth Factor*.

* *P ≤ 0.05*;

** *P < 0.01*;

*** *P < 0.001 with statistically significant changes highlighted bold*.

**Figure 4:**
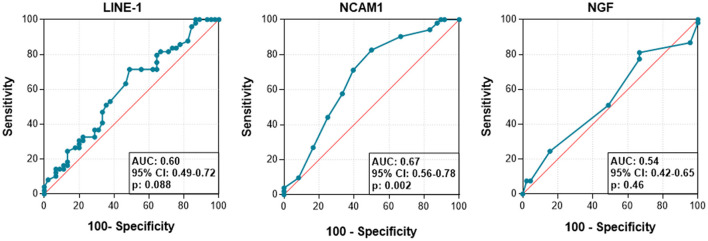
Receiver operator characteristic curves (ROC) and area under the curve (AUC) values to differentiate ASD from healthy condition according to the methylation status of each target.

In the ROC curve, the AUC was calculated to explore differences in *LINE-1, NCAM1* and/or *NGF* methylation between ASD and healthy status (Figure 4). The AUC for *NCAM1* was 0.67 (*p* = 0.02). However, for *LINE-1* and *NGF*, the values resulted not optimal to discriminate between ASD and healthy conditions.

## Discussion

In the present work we report results from a global and local DNA methylation analysis of a pediatric sample with ASD compared to a normally developing children subgroup. ASD is a neurodevelopmental disorder that presumably affects multiple areas of the brain, including the frontal, temporal, and occipital cortex, as well as the cerebellum. Since brain tissue cannot be collected from live individuals, a more accessible tissue sample has been suggested, such as peripheral blood. High correlations have been found between methylation levels of specific genes in the blood and brain regions in children with neurodevelopmental disorders and controls (6, 35, 36).

DNA methylation in non-coding regions, including *LINE-1* retrotransposons, which constitutes a substantial portion of the human genome, has been very little investigated in the context of ASD. The first report of an increase in *LINE-1* expression in the autism cerebellum was published by Shpyleva et al. (34). These authors carried out a methylated DNA immunoprecipitation (MeDIP) analysis of cytosine-5 methylation in *LINE-1*, obtaining a downward trend of *LINE-1* methylation in ASD, although this difference was not statistically significant, possibly due to heterogeneity within the ASD population. However, they also observed significantly less binding of repressive MeCP2 and histone H3K9me3 to *LINE-1* sequences in the autistic cerebellum, suggesting that relaxation of epigenetic repression may have aided in increased expression. Moreover, since the results were obtained from postmortem brain samples, it could not be determined whether these observations were functionally related to the etiology or pathophysiology of autism.

A recent study with 36 ASD subjects and 20 unaffected individuals showed that the *LINE-1* methylation levels were significantly reduced in ASD individuals with severe language impairment and were inversely correlated with the transcript level (37). Nevertheless, this study was carried out using lymphoblastoid cell lines derived from peripheral mononuclear cells. Here, we report an analysis of *LINE-1* methylation status using blood samples from 50 ASD patients and 45 control individuals. Our results show a slight decrease in the levels of *LINE-1* methylation in the ASD group, compared to the normal neurodevelopmental healthy group, with the following mean values: 73.5% in ASD and 74.2% in controls. Although these differences in the pattern of *LINE-1* methylation between the ASD group and the control group are not statistically significant, they are higher than those observed in relevant studies also carried out by pyrosequencing in other neurodegenerative diseases. In this regard, Bollati et al. (38) evaluated methylation of *ALU, LINE-1* (same CpGs fragment than in the present study), and *SAT-*α sequences in blood samples from 43 patients with Alzheimer's disease (AD) and 38 healthy donors. *LINE-1* methylation increased in AD patients, compared to healthy volunteers, obtaining the following mean methylation values: AD 83.6% and volunteers 83.1% (*p*-value: 0.05).

Furthermore, our study focused on a *LINE-1* fragment containing 5 CpG positions for analysis. Remarkably, we observed a statistically significant decrease in methylation levels at position 2 of the *LINE-1* analyzed fragment, which is then reflected in the subgroup of children with mental regression. Thus, while the mean CpG 2 methylation value of *LINE-1* in healthy controls is 72.97%, it falls to 71.15% in the subgroup of AMR children (*p*-value: 0.047). This result should be interpreted as a strong indicator for expanding the *LINE-1* study region. Therefore, a more in-depth future study of *LINE-1* methylation patterns in patients with ASD and healthy controls with an enlarged analyzed region is needed.

Quantification of DNA methylation in candidate gene promoters using different samples (blood, specific regions in post-mortem brain) from patients with ASD has identified differentially methylated regions (DMR) in OXTR, SHANK3, UBE3A, and MECP2 at CpG sites of specific promoters (5–9). Increasing evidence suggests that growth factors modulate motor, emotional and cognitive functions, which may account for the clinical manifestations of several disorders (39). NGF belongs to the neurotrophin family and is considered a key regulator of the development, differentiation, survival and regeneration of nerve cells. NGF is present mainly in highly functional brain regions. Previous studies from our group of the same cohort of ASD children as this study, as well as other groups of children with this disorder, have shown increased levels of *NGF* in children with ASD (27). Moreover, in this disorder, different subgroups considering the presence or absence of neurodevelopmental regression have been reported (40, 41). Our results seem to support these notions, since we found different *NGF* methylation patterns in both subgroups, AMR and ANMR. In this regard, approximately half of both children diagnosed with ASD (47%) and typically developing children (53%) have lower-than-mean levels of *NGF* methylation.

These results are consistent with those previously observed by our group, in which *NGF* was detected in < 50% of the children with ASD and typically developing children (27). More specifically, while only 39% of the ANMR subgroup showed increased levels of *NGF* methylation, this value rose to more than 60% in the AMR subgroup, suggesting a possible higher expression of *NGF* in the first subgroup. This is precisely what we showed in our previously published results, using samples from the same individuals, where the ANMR subgroup exhibited higher *NGF* levels than the typically developing children (27), making even more plausible the suggestion that these observations might indicate a disturbance in neuronal development, and pointing to *NGF* as a possible biomarker in autism disorder.

*NCAM1* is a glycoprotein mainly expressed on the surface of nerve cells, in the central and peripheral nervous tissue of vertebrates, and acts as an adhesion molecule between cells and their extracellular environment (14, 41). It plays a critical role in the developmental and plasticity pathways of the nervous system (8). *NCAM1* may accumulate in presynaptic and postsynaptic membranes and it has been associated with behavioral phenotypes in ASD children (42). Changes in DNA methylation have not yet been described at the *NCAM1* level. In the present study, we reported that when compared to the typically developing children subgroup, higher levels of *NCAM1* methylation were observed in children with ASD, without differentiation between the AMR and the ANMR subgroups. Interestingly, and in agreement with these data, our previous studies found lower plasma levels of *NCAM1* in the same subgroup of ASD children without neurodevelopmental regression, compared to the levels in the subgroup of typically developing children (27). *NCAM1* could be suggested as a possible biomarker, capable of contributing to the diagnostic process of the autistic disorder.

In conclusion, given the high heterogeneity in ASD, it is essential to know the biological factors that trigger this disorder to understand the behavioral variability of the patients. Identification of epigenetic biomarkers based on changes in DNA methylation could help to clarify the pathophysiology of autism, facilitating its diagnosis and prognosis. To date, a consensus has not yet been generated on the gene's specific methylation signature for autism. In this study, we provide new clues about the epigenetic changes that occur in the autistic disorders as well as a clear differentiation in DNA methylation between the ASD subgroups, according to the presence or absence of mental regression. Thus, we have reported a differentially methylated CpG position in the *LINE-1* retrotransposon between healthy children and children with ASD. In addition, we have pointed two candidate genes, *NGF* and *NCAM1*, as potential epigenetic biomarkers that would facilitate the diagnosis of the disorder. Finally, the findings of this study are in line with previous research and reinforce our understanding of this disorder. However, we consider that further investigation is necessary in this line of research.

## Data Availability Statement

The raw data supporting the conclusions of this article will be made available by the authors, without undue reservation.

## Ethics Statement

The studies involving human participants were reviewed and approved by Ethics Committee of Investigation of Cordoba. Written informed consent to participate in this study was provided by the participants' legal guardian/next of kin.

## Author Contributions

MVG-O, TM-R, MT-A, TR-A, RRA, and JP-N were involved in planning and supervised the work. AG-F, KF-R, PM-B, and MT-A processed the clinical data. MVG-O and TM-R processed the experimental data and performed the analysis. MVG-O, TR-A, MG-C, MT-A, and JP-N drafted the manuscript. All authors discussed the results and commented on the manuscript.

## Conflict of Interest

The authors declare that the research was conducted in the absence of any commercial or financial relationships that could be construed as a potential conflict of interest.

## Publisher's Note

All claims expressed in this article are solely those of the authors and do not necessarily represent those of their affiliated organizations, or those of the publisher, the editors and the reviewers. Any product that may be evaluated in this article, or claim that may be made by its manufacturer, is not guaranteed or endorsed by the publisher.

## References

[1] Menezo YJ Elder K Dale B. Link between increased prevalence of autism spectrum disorder syndromes and oxidative stress, DNA methylation, and imprinting: the impact of the environment. JAMA Pediatr. (2015) 169:1066–7. 10.1001/jamapediatrics.2015.2125 26414354

[2] Fombonne E. Epidemiology of autistic disorder and other pervasive developmental disorders. J Clin Psychiatry. (2005) 66(Suppl. 10):3–8. 16401144

[3] Yoon SH Choi J Lee WJ Do JT. Genetic and epigenetic etiology underlying autism spectrum disorder. J Clin Med. (2020) 9:966. 10.3390/jcm9040966 32244359 PMC7230567

[4] Wen Z Cheng TL Li GZ Wen Z Cheng TL Li GZ. Identification of autism-related MECP2 mutations by whole-exome sequencing and functional validation. Mol Autism. (2017) 8:43. 10.1186/s13229-017-0157-5 28785396 PMC5543534

[5] Elagoz Yuksel M Yuceturk B Faruk Karatas O Ozen M Dogangun B. The altered promoter methylation of oxytocin receptor gene in autism. J Neurogenet. (2016) 30:280–4. 10.1080/01677063.2016.1202951 27309964

[6] Gregory SG Connelly JJ Towers AJ Johnson J Biscocho D Markunas CA. Genomic and epigenetic evidence for oxytocin receptor deficiency in autism. BMC Med. (2009) 7:62. 10.1186/1741-7015-7-62 19845972 PMC2774338

[7] Jiang YH Sahoo T Michaelis RC Bercovich D Bressler J Kashork CD. A mixed epigenetic/genetic model for oligogenic inheritance of autism with a limited role for UBE3A. Am J Med Genet A. (2004) 131:1–10. 10.1002/ajmg.a.30297 15389703

[8] Nagarajan RP Hogart AR Gwye Y Martin MR LaSalle JM. Reduced MeCP2 expression is frequent in autism frontal cortex and correlates with aberrant MECP2 promoter methylation. Epigenetics. (2006) 1:e1–11. 10.4161/epi.1.4.3514 17486179 PMC1866172

[9] Zhu L Wang X Li XL Towers A Cao X Wang P. Epigenetic dysregulation of SHANK3 in brain tissues from individuals with autism spectrum disorders. Hum Mol Genet. (2014) 23:1563–78. 10.1093/hmg/ddt547 24186872 PMC3929093

[10] Tremblay MW Jiang YH. DNA methylation and susceptibility to autism spectrum disorder. Annu Rev Med. (2019) 27:151–66. 10.1146/annurev-med-120417-091431 30691368 PMC6597259

[11] Bailey A Le Couteur A Gottesman I Bolton P Simonoff E Yuzda E. Autism as a strongly genetic disorder: evidence from a British twin study. Psychol Med. (1995) 25:63–77. 10.1017/S0033291700028099 7792363

[12] Goldberg AD Allis CD Bernstein E. Epigenetics: a landscape takes shape. Cell. (2007) 23:635–8. 10.1016/j.cell.2007.02.006 17320500

[13] Jones PA. Functions of DNA methylation: islands, start sites, gene bodies and beyond. Nat Rev Genet. (2012) 29:484–92. 10.1038/nrg3230 22641018

[14] Vogel Ciernia A LaSalle J. The landscape of DNA methylation amid a perfect storm of autism aetiologies. Nat Rev Neurosci. (2016) 17:411–23. 10.1038/nrn.2016.41 27150399 PMC4966286

[15] Grayson DR Guidotti A. Merging data from genetic and epigenetic approaches to better understand autistic spectrum disorder. Epigenomics. (2016) 8:85–104. 10.2217/epi.15.92 26551091 PMC4864049

[16] Keil KP Lein PJ. DNA methylation: a mechanism linking environmental chemical exposures to risk of autism spectrum disorders. Environ Epigenet. (2016) 2:dvv012. 10.1093/eep/dvv012 27158529 PMC4856164

[17] Loke YJ Hannan AJ Craig JM. The role of epigenetic change in autism spectrum disorders. Front Neurol. (2015) 26:107. 10.3389/fneur.2015.00107 26074864 PMC4443738

[18] Siu MT Weksberg R. Epigenetics of autism spectrum disorder. Adv Exp Med Biol. (2017) 978:63–90. 10.1007/978-3-319-53889-1_4 28523541

[19] Wiśniowiecka-Kowalnik B Nowakowska BA. Genetics and epigenetics of autism spectrum disorder-current evidence in the field. J Appl Genet. (2019) 60:37–47. 10.1007/s13353-018-00480-w 30627967 PMC6373410

[20] Ladd-Acosta C Hansen KD Briem E Fallin MD Kaufmann WE Feinberg AP. Common DNA methylation alterations in multiple brain regions in autism. Mol Psychiatry. (2014) 19:862–71. 10.1038/mp.2013.114 23999529 PMC4184909

[21] Andrews SV Ellis SE Bakulski KM Sheppard B Croen LA Hertz-Picciotto I. Cross-tissue integration of genetic and epigenetic data offers insight into autism spectrum disorder. Nat Commun. (2017) 24:1011. 10.1101/091330 29066808 PMC5654961

[22] Andrews SV Sheppard B Windham GC Schieve LA Schendel DE Croen LA. Case-control meta-analysis of blood DNA methylation and autism spectrum disorder. Mol Autism. (2018) 28:40. 10.1101/320622 29988321 PMC6022498

[23] De Rubeis S He X Goldberg AP Poultney CS Samocha K Cicek AE. Synaptic, transcriptional and chromatin genes disrupted in autism. Nature. (2014) 13:209–15. 10.1038/nature13772 25363760 PMC4402723

[24] Cordaux R Batzer MA. The impact of retrotransposons on human genome evolution. Nat Rev Genet. (2009) 10:691–703. 10.1038/nrg2640 19763152 PMC2884099

[25] Han JS Szak ST Boeke JD. Transcriptional disruption by the L1 retrotransposon and implications for mammalian transcriptomes. Nature. (2004) 20:268–74. 10.1038/nature02536 15152245

[26] Kitkumthorn N Mutirangura A. Long interspersed nuclear element-1 hypomethylation in cancer: biology and clinical applications. Clin Epigenetics. (2011) 2:315–30. 10.1007/s13148-011-0032-8 22704344 PMC3365388

[27] Gomez-Fernandez A de la Torre-Aguilar MJ Gil-Campos M Flores-Rojas K Cruz-Rico MD Martin-Borreguero P. Children with autism spectrum disorder with regression exhibit a different profile in plasma cytokines and adhesion molecules compared to children without such regression. Front Pediatr. (2018) 26:264. 10.3389/fped.2018.00264 30320048 PMC6169449

[28] APA. DSM-V. (2013). Available online at: http://www.dsm5.org/Pages/Default.aspx

[29] Beck CR Garcia-Perez JL Badge RM Moran JV. LINE-1 elements in structural variation and disease. Annu Rev Genomics Hum Genet. (2011) 12:187–215. 10.1146/annurev-genom-082509-141802 21801021 PMC4124830

[30] Plioplys AV Hemmens SE Regan CM. Expression of a neural cell adhesion molecule serum fragment is depressed in autism. J Neuropsychiatry Clin Neurosci. (1990) 2:413–7. 10.1176/jnp.2.4.413 2136394

[31] Purcell AE Rocco MM Lenhart JA Hyder K Zimmerman AW Pevsner J. Assessment of neural cell adhesion molecule (NCAM) in autistic serum and postmortem brain. J Autism Dev Disord. (2001) 31:183–94. 10.1023/A:1010751232295 11450817

[32] Guney E Ceylan MF Kara M Tekin N Goker Z Senses Dinc G. Serum nerve growth factor (NGF) levels in children with attention deficit/hyperactivity disorder (ADHD). Neurosci Lett. (2014) 7:107–11. 10.1016/j.neulet.2013.12.026 24361544

[33] Anton L Brown AG Bartolomei MS Elovitz MA. Differential methylation of genes associated with cell adhesion in preeclamptic placentas. PLoS ONE. (2014) 25:e100148. 10.1371/journal.pone.0100148 24963923 PMC4070941

[34] Shpyleva S Melnyk S Pavliv O Pogribny I Jill James S. Overexpression of LINE-1 retrotransposons in autism brain. Mol Neurobiol. (2018) 55:1740–9. 10.1007/s12035-017-0421-x 28220356

[35] Murphy BC O'Reilly RL Singh SM. Site-specific cytosine methylation in S-COMT promoter in 31 brain regions with implications for studies involving schizophrenia. Am J Med Genet B Neuropsychiatr Genet. (2005) 5:37–42. 10.1002/ajmg.b.30134 15635661

[36] Kaminsky Z Tochigi M Jia P Pal M Mill J Kwan A. A multi-tissue analysis identifies HLA complex group 9 gene methylation differences in bipolar disorder. Mol Psychiatry. (2012) 17:728–40. 10.1038/mp.2011.64 21647149

[37] Tangsuwansri C Saeliw T Thongkorn S Chonchaiya W Suphapeetiporn K Mutirangura A. Investigation of epigenetic regulatory networks associated with autism spectrum disorder (ASD) by integrated global LINE-1 methylation and gene expression profiling analyses. PLoS ONE. (2018) 23:13:e0201071. 10.1371/journal.pone.0201071 30036398 PMC6056057

[38] Bollati V Galimberti D Pergoli L Dalla Valle E Barretta F Cortini F. DNA methylation in repetitive elements and Alzheimer disease. Brain Behav Immun. (2011) 25:1078–83. 10.1016/j.bbi.2011.01.017 21296655 PMC3742099

[39] Galvez-Contreras AY Campos-Ordonez T Gonzalez-Castaneda RE Gonzalez-Perez O. Alterations of growth factors in autism and attention-deficit/hyperactivity disorder. Front Psychiatry. (2017) 8:126. 10.3389/fpsyt.2017.00126 28751869 PMC5507945

[40] Kern JK Geier DA Geier MR. Evaluation of regression in autism spectrum disorder based on parental reports. N Am J Med Sci. (2014) 6:41–7. 10.4103/1947-2714.125867 24678477 PMC3938873

[41] Xi CY Hua TY Zhao YJ Liu XM. Characteristics of developmental regression in autistic children. Zhongguo Dang Dai Er Ke Za Zhi. (2010) 12:781–3. 20959041

[42] Yang X Zou M Pang X Liang S Sun C Wang J. The association between NCAM1 levels and behavioral phenotypes in children with autism spectrum disorder. Behav Brain Res. (2019) 1:234–8. 10.1016/j.bbr.2018.11.012 30423390

