# Supplementary material for: Analysis of Global and Local DNA Methylation Patterns in Blood Samples of Patients With Autism Spectrum Disorder
Source: Front Pediatr. 2021 Oct 5;9:685310. doi: 10.3389/fped.2021.685310 (PMC8524094; doi:10.3389/fped.2021.685310)
Supplement: Supplementary file 1 [file Data_Sheet_1.docx]

**SUPPLEMENTARY TABLES**

**Supplementary Table 1.** Pyrosequencing results showing mean values ± standard error of the mean (SEM) values obtained for LINE-1 in ASD group, ANMR and AMR subgroups compared to healthy control group.

|  | **LINE-1** | | | |
| --- | --- | --- | --- | --- |
|  | **Control** | **ASD** | **ANMR** | **AMR** |
| **All CpG** | 74,56 ± 0,45 | 73,45 ± 0,43 | 73,51 ± 0,62 | 73,61 ± 0,55 |
| **CpG site 1** | 80,15 ± 0,54 | 79,60 ± 0,48 | 79,60 ± 0,62 | 79,60 ± 0,79 |
| **CpG site 2** | 72,98 ± 0,52 | 71,48 ± 0,49 | 71,48 ± 0,73 | 71,16 ± 0,60 |
| **CpG site 3** | 73,98 ± 0,42 | 72,82 ± 0,41 | 72,63 ± 0,60 | 73,10 ± 0,51 |
| **CpG site 4** | 81,37 ± 0,41 | 80,70 ± 0,36 | 80,53 ± 0,54 | 80,95 ± 0,41 |
| **CpG site 5** | 63,84 ± 0,46 | 62,77 ± 0,50 | 62,65 ± 0,71 | 62,95 ± 0,69 |

ASD: autism spectrum disorders; AMR: autism mental regression; ANMR: autism non-mental regression

**Supplementary Table 2.** Pyrosequencing results showing mean values ± standard error of the mean (SEM) values obtained for NCAM1 in ASD group, ANMR and AMR subgroups compared to healthy control group.

|  | **NCAM1** | | | |
| --- | --- | --- | --- | --- |
|  | **Control** | **ASD** | **ANMR** | **AMR** |
| **All CpG** | 88,46 ± 0,226 | 89,42 ± 0,173 | 89,42 ± 0,227 | 89,43 ± 0,272 |
| **CpG site 1** | 96,83 ± 0,225 | 98,24 ± 0,177 | 98,15 ± 0,224 | 98,40 ± 0,294 |
| **CpG site 2** | 80,08 ± 0,311 | 80,51 ± 0,31 | 80,54 ± 0,404 | 80,45 ± 0,495 |

ASD: autism spectrum disorders; AMR: autism mental regression; ANMR: autism non-mental regression; NCAM1: Neural Cell Adhesion Molecule

**Supplementary Table 3.** Pyrosequencing results showing mean values ± standard error of the mean values obtained for NGF in ASD group, ANMR and AMR subgroups compared to healthy control group.

|  |  | **NGF** | | | |
| --- | --- | --- | --- | --- | --- |
|  |  | **Control** | **ASD** | **ANMR** | **AMR** |
| **All CpG** |  | 2,76 ± 0,06 | 2,80 ± 0,07 | 2,68 ± 0,08 | 3,04 ± 0,09 |
| **CpG site 1** |  | 2,11 ± 0,07 | 2,24 ± 0,07 | 2,18 ± 0,07 | 2,35 ± 0,15 |
| **CpG site 2** |  | 1,66 ± 0,07 | 1,62 ± 0,07 | 1,55 ± 0,09 | 1,75 ± 0,10 |
| **CpG site 3** |  | 4,49 ± 0,08 | 5,07 ± 0,10 | 5,00 ± 0,12 | 5,16 ± 0,17 |

ASD: autism spectrum disorders; AMR: autism mental regression; ANMR: autism non-mental regression; NGF: Nerve Growth Factor

**Supplementary Table 4**. Spearman´s correlation coefficient between methylation status of LINE-1 with cognitive and behavioral measures in children with autism spectrum disorders

|  |  | **NGF** | | | | | | **NCAM** | | | |
| --- | --- | --- | --- | --- | --- | --- | --- | --- | --- | --- | --- |
|  |  | **CpG site 1** | | **CpG site** 2 | | **CpG site 3** | | **CpG site 1** | | **CpG site 2** | |
|  |  | r | p | r | p | r | p | r | p | r | p |
| **CARS** |  | 0.247 | 0.075 | 0.089 | 0.526 | -0.09 | 0.991 | 0.001 | 0.999 | -0.06 | 0.619 |
| **Battelle** | Personal | -0.21 | 0.132 | 0.000 | 1 | -0.06 | 0.663 | -0.05 | 0.695 | 0.126 | 0.367 |
|  | Adaptative | -0.19 | 0.176 | 0.04 | 0.774 | -0.07 | 0.622 | -0.09 | 0.982 | 0.115 | 0.411 |
|  | Gross Motor | -0.19 | 0.428 | -0.09 | 0.897 | 0.108 | 0.447 | -0.14 | 0.315 | 0.213 | 0.126 |
|  | Fine Motor | -0.11 | 0.409 | -0.04 | 0.745 | 0.04 | 0.729 | -0.17 | 0.199 | -0.05 | 0.679 |
|  | Receptive | -0.17 | 0.211 | 0.041 | 0.774 | -0.09 | 0.872 | -0.12 | 0.39 | -0.06 | 0.967 |
|  | Expressive | -0.19 | 0.160 | 0.125 | 0.377 | -0.02 | 0.887 | 0.029 | 0.837 | -0.02 | 0.839 |
|  | Communication | -0.29 | 0.152 | 0.087 | 0.540 | -0.04 | 0.755 | -0.04 | 0.757 | -0.05 | 0.697 |
|  | Total | -0.19 | 0.162 | 0.01 | 0.904 | 0.01 | 0.924 | -0.06 | 0.632 | 0.06 | 0.635 |
| **PDDBI** | Sensory | 0.008 | 0.959 | -0.31 | 0.028 | -0.11 | 0.424 | -0.18 | 0.213 | -0.09 | 0.513 |
|  | Ritual | 0.189 | 0.198 | -0.05 | 0.71 | -0.05 | 0.731 | -0.15 | 0.280 | -0.07 | 0.597 |
|  | Social Pragmatic Problems | 0.121 | 0.417 | -0.03 | 0.802 | -0.04 | 0.760 | -0.03 | 0.832 | 0.167 | 0.257 |
|  | Semantic Pragmatic Problems | 0.02 | 0.874 | 0.178 | 0.222 | 0.08 | 0.548 | 0.021 | 0.884 | -0.11 | 0.428 |
|  | Social Aproach Behaviours | -0.06 | 0.641 | -0.04 | 0.772 | 0.08 | 0.585 | -0.19 | 0.181 | -0.05 | 0.707 |
|  | Expresive Language | -0.23 | 0.111 | 0.03 | 0.804 | -0.01 | 0.941 | -0.13 | 0.336 | -0.08 | 0.566 |
|  | Score Autism | 0.246 | 0.08 | -0.11 | 0.480 | -0.14 | 0.314 | -0.03 | 0.814 | 0.04 | 0.753 |

CARS: childhood autism rating scale; PDDBI: Pervasive Developmental Disorders Behaviour Inventory;

NGF: Nerve Growth Factor; NCAM1: Neural Cell Adhesion Molecule
